# Species-level profiling of *Landoltia punctata* (duckweed) microbiome under nutrient stress using full-length 16S rRNA sequencing

**DOI:** 10.7717/peerj.20648

**Published:** 2026-02-06

**Authors:** Chakrit Bunyoo, Juthaporn Phonmakham, Masaaki Morikawa, Arinthip Thamchaipenet

**Affiliations:** 1 Department of Genetics, Faculty of Science, Kasetsart University, Bangkok, Thailand; 2 Duckweed Holobiont Resource & Research Center (DHbRC), Kasetsart University, Bangkok, Thailand; 3 Central Scientific Instrument Management Laboratory, Bureau for Research and Innovation Management, Chulabhorn Royal Academy, Bangkok, Thailand; 4 Omics Center for Agriculture, Bioresource, Food and Health Kasetsart University (OmiKU), Kasetsart University, Bangkok, Thailand; 5 Graduate School of Environmental Science, Hokkaido University, Sapporo, Japan

**Keywords:** Duckweed microbiome, *Landoltia punctata*, Metagenome, Full-length 16S rRNA gene, Nutrient-deficient stress

## Abstract

Duckweed is a rapidly-growing aquatic plant utilized as food/feed and for wastewater remediation. It coexists with complex microbial communities that play crucial roles in its growth and capability for phytoremediation. In a previous study, microbiomes associated with four duckweed species (*Spirodela polyrhiza*, *Landoltia punctata*, *Lemna aequinoctialis*, and *Wolffia globosa*) grown under natural and nutrient-deficient conditions, were investigated using V3V4 16S rRNA sequencing. However, species-level classification was not achieved due to the partial 16S rRNA sequences obtained, restricting the selection of potential microbial species for further application. In this study, *L. punctata* samples from the previous work were investigated further by employing full-length 16S rRNA sequencing. A total of 31 predominant microbial species were identified. Under stress, the proportion of Proteobacteria increased significantly, along with potentially beneficial bacteria such as *Roseateles depolymerans*, *Pelomonas saccharophila*, *Acidovorax temperans*, *Ensifer adhaerens* and *Rhizobium straminoryzae*. Functional metagenomic predictions suggest that associated microbes adapt to stressors and may confer benefits to duckweed, including pathways related to host adhesion, biofilm formation, microbial growth modulation, and co-factors and vitamin biosynthesis. Furthermore, the study demonstrates both the advantages and limitations of full-length 16S rRNA amplicon sequencing. The findings provide more insight into *L. punctata* microbiomes at species-level, facilitating establishment of stable, beneficial microbial communities for duckweed applications. Ongoing investigations aim to isolate key microbial species from *L. punctata* and validate their roles through co-cultivation, along with establishing potential synthetic microbial communities based on the metagenomic findings.

## Introduction

Duckweed (Lemnaceae family) is a free-floating aquatic plant that thrives in both tropical and temperate regions (Landolt & Kandeler, 1987). It is comprised of five genera: *Spirodela*, *Landoltia*, *Lemna*, *Wolffiella*, and *Wolffia*, with a total of 36 species identified (Bog, Appenroth & Sree, 2019). Duckweed has a simple structure consisting of roots and leaf-like organs called fronds. For some genera, such as *Wolffiella* and *Wolffia* the fronds are rootless (Yang et al., 2021). Fast-growing duckweed has a high content of protein and starch, while maintaining low levels of cellulose and lignin accumulation (Xu et al., 2011; Appenroth et al., 2018). These characteristics position duckweed as a potential source of alternative protein for food and feed, and source of starch for industrial purposes such as biofuel (Xu et al., 2011; Ziegler et al., 2015; Appenroth et al., 2017; Oláh, Appenroth & Sree, 2023; Kang et al., 2025). Duckweed can utilize excess nitrogen (N) or phosphorus (P) from water sources efficiently for biomass production (Zhou & Borisjuk, 2019). The utilization of duckweed for wastewater treatment has shown considerable potential due to its effectiveness and low cost (Zimmo, van der Steen & Gijzen, 2004; Zhou & Borisjuk, 2019; Toyama et al., 2024).

Duckweed lives with associated microbes (Acosta et al., 2020). Duckweed-associated bacteria (DAB) provide several benefits to duckweed, including growth promotion, bioremediation, and tolerance to environmental stress (Suzuki et al., 2014; Chen et al., 2019; Ishizawa et al., 2020a; Boonmak et al., 2023). Metagenomic data further suggest that significant microbial populations within duckweed ecosystems tend to display similarity across different terrestrial plants (Acosta et al., 2020). These cohabiting DAB have been previously identified as common plant-associated bacteria, such as members of the genera *Acinetobacter*, *Acidovorax*, *Pseudomonas*, *Rhizobium*, *Pelomonas*, *Roseateles*, and *Novosphingobium* (Acosta et al., 2020; Bunyoo et al., 2022; Inoue et al., 2022; Boonmak et al., 2023). Recent studies have validated the beneficial roles of microbiomes to duckweeds by providing benefits while interacting with their host mutualistically (Ishizawa et al., 2017; Laurich et al., 2024). Furthermore, co-cultivation as synthetic microbial communities (SynCom) are more effective and result in higher production of biomass than co-cultivation with a single strain (Ishizawa et al., 2020b; Laurich et al., 2024; Thanh Phaṃ et al., 2025). Therefore, a deep understanding of duckweed-associated microbial communities and their functions will be critical for establishing beneficial SynCom.

In a previous study, the microbiome associated with four species of duckweed including *Spirodela polyrhiza*, *Landoltia punctata*, *Lemna aequinoctialis*, and *Wolffia globosa*, was grown under natural and nutrient-deficient conditions and investigated using V3-V4 16S rRNA amplicon sequencing (Bunyoo et al., 2022). The findings revealed potentially beneficial core microbiomes that persisted in association with duckweed under stress, including members of the genera *Allorhizobium*-*Neorhizobium*-*Pararhizobium*-*Rhizobium*, *Pelomonas*, *Roseateles*, and *Novosphingobium*. However, taxonomic classification in that study was limited at the genus level due to the use of partial 16S rRNA sequencing (Bunyoo et al., 2022). This limitation also constrains the ability to select and design SynCom for further improving the duckweed traits. Although, partial 16S rRNA sequencing based on short-read technology is widely used in metagenomic studies, it introduces biases and delivers inefficient data to verify taxonomy at species levels, thereby restricting microbial ecological studies (Guo et al., 2013; Mizrahi-Man, Davenport & Gilad, 2013; Hrovat et al., 2024). Alternatively, full-length 16S rRNA gene (FL-16S) sequencing allows significant advantages of species level identification (Schloss et al., 2016; Earl et al., 2018; Hale et al., 2024). Nevertheless, only a few studies of FL-16S sequencing in aquatic plant microbiomes have been undertaken.

Here, the bacterial communities associated with naturally-growing and stressed *L. punctata,* previously identified as the most resilient species, were investigated further using FL-16S sequencing to compare taxonomic resolution with those of previous investigations using V3V4-16S sequencing (Bunyoo et al., 2022). Subsequently, the species identified will be evaluated for their potential function towards *L. punctata*. The findings will contribute to a better understanding of duckweed microbiomes, aiding in the selection of beneficial species based on metagenomic data for the establishment of stable SynCom to improve duckweed traits.

## Materials & Methods

### Samples collection and DNA isolation

The same DNA samples of *Landoltia punctata* and the ambient (surrounding) water from the previous study (Bunyoo et al., 2022) were used in this study. Each sample type included five replicates, which were investigated further for microbial communities at the species level. The sample collections, cultivation conditions, and DNA extraction method were performed as described previously (Bunyoo et al., 2022). Briefly, duckweed samples were rinsed three times in sterilized water before storage in DNA/RNA shield™ (Zymo Research Corp, Irvine, CA, USA) at −80 °C until used. The water samples were first passed through sterilized Whatman filter paper, grade 4 (20–25 µm), followed by filtering through a Whatman WME membrane (0.2 µm) to capture microbial communities. The filters were cut into small pieces and preserved as described above.

Approximately five grams (fresh weight) of natural *L. punctata* was grown in sterilized distilled water (nutrient-deficient condition) in clean glass containers at 25 °C under a photoperiod of 12 h with a light intensity of 50 µmol m^−2^ s^−1^. After 14 days of cultivation, duckweed samples were harvested and preserved as described above.

DNA from *L. punctata* and ambient water samples was extracted using ZymoBIOMICS™ DNA Miniprep Kit (Zymo Research Corp, Irvine, CA, USA) following the manufacturer’s protocol. A total of 15 DNA samples were analyzed: *L. punctata* under natural conditions (*n* = 5), *L. punctata* under 2-week nutrient deficient conditions (*n* = 5), and ambient water samples (*n* = 5). ZymoBIOMICS™ Microbial Community DNA Standard (Mock) samples (Lot no. ZRC 193008) consisting of eight valid species: *Bacillus subtilis*, *Enterococcus faecalis*, *Escherichia coli*, *Lactobacillus fermentum*, *Listeria monocytogenes*, *Pseudomonas aeruginosa*, *Salmonella enterica*, and *Staphylococcus aureus* were included as a control for each sequencing run (*n* = 4).

### Polymerase chain reaction amplification, library preparation, and PacBio sequencing

Library preparation for the FL-16S gene was carried out by two-step polymerase chain reaction (PCR) amplification. The first-round amplification reaction was performed using a pair of 5′  block primers, 27F (5AmMC6/gcagtcgaacatgtagctgactcaggtcacAGRGTTYGATYMTGGCTCAG)and 1492R  (5AmMC6/tggatcacttgtgcaagcatcacatcgtagRGYTACCTTGTTACGACTT),provided by PacBio (Pacific Bioscience, Menlo Park, CA, USA). The PCR reaction (25 µl) consisted of 2.5 ng DNA template, 0.2 µM of each primer, 0.4 U Phusion High-Fidelity DNA Polymerase (Thermo Fisher Scientific, Watham, MA, USA), 1x Phusion HF buffer, 0.2 mM dNTPs in DNase free water. Amplification was for 20 cycles at 95 °C for 30 s, 57 °C for 30 s, and 72 °C for 90 s. Second-round PCR was performed using PacBio Barcoded Universal Primers (https://doi.org/10.6084/m9.figshare.31076803) with one nanogram of the first round PCR product under the amplification reaction conditions described above.

The secondary PCR amplicons were purified using AMPure PB (PacBio; Pacific Bioscience) and quantified using a Qubit 2.0 Fluorometer and Qubit dsDNA BR Assay Kit (Thermo Fisher Scientific). Purified amplicons were pooled in equimolar concentrations so that each pool contained barcoded amplicons from 4–5 samples. The SMRTbell libraries were prepared using a SMRTbell Express Template Prep Kit 2.0 (PacBio; Pacific Bioscience) according to the manufacturer’s instructions. The libraries were sequenced on a PacBio Sequel System (PacBio; Pacific Bioscience) using a Sequel sequencing kit 3.0 and SMRTcell 1M v3 LR (PacBio; Pacific Bioscience) with 10-hour movie time. The raw sequence data generated in this study were deposited at the National Center for Biotechnology Information (NCBI) under BioProject accession number PRJNA1016047.

### PacBio data processing and metagenomic analysis

PacBio raw reads were processed using SMRT Analysis software version 9.0 (PacBio; Pacific Bioscience, USA) to obtain demultiplexed consensus sequences with a minimum of three full passes. Circular consensus sequencing (CCS) reads were processed using the package DADA2 (Callahan et al., 2016) in R (version 4.1.1; R Core Team, 2021). The primer sequences and sequence qualities (minQ = 2, minLen = 1,200, maxLen = 1,600, maxN = 0, maxEE = 2) were filtered prior to downstream analysis. The remaining sequences were dereplicated and clustered into amplicon sequence variants (ASVs) with default parameters. *De novo* chimera filtering was performed using removeBimeraDenovo in the DADA2 package. ASVs with a total read count of fewer than five across all samples were filtered out prior to downstream analysis. The rarefaction curve was analyzed to evaluate whether sequencing depth was sufficient to capture the microbial diversity within a sample. A taxonomic classification was assigned to representative sequences in each ASV using a naive Bayesian classifier method (minBoot = 80) and DADA2-formatted Silva version 138.1 (Quast et al., 2013; https://benjjneb.github.io/dada2/training.html). For species detection, the ASV sequences were aligned to GenBank 16S rRNA reference sequences (type strain) using BLASTN (Camacho et al., 2009) with percentage of identity above 97 and *E*-value cut-off at 1^e−10^. Any query sequence that returned more than two hits belonging to the same species with identical *E*-value and percentage of identity was considered as unclassifiable in species ranking. The feature table was imported to the phyloseq R package (McMurdie & Holmes, 2013). The chloroplast and mitochondria reads were removed from the final feature table.

### Mock bacterial community analysis

To evaluate the sequencing accuracy of PacBio CCS data, representative sequences of each ASV in the mock samples were aligned using BLASTN with the mock 16S/18S reference sequences provided by the manufacturer (https://doi.org/10.6084/m9.figshare.31076803). The accuracy of the sequences was determined by calculating the percentage of identity with the reference sequences. The relative abundance (%) of each detected species was compared to the theoretical relative abundance provided by the manufacturer (https://files.zymoresearch.com/protocols/_d6300_zymobiomics_microbial_community_standard.pdf).

### Prediction of metagenome functions

To predict metabolic pathways and functions of microbiomes in each group, the ASVs table was analyzed using PICRUSt2 (Douglas et al., 2020). The predicted metabolic profiles were categorized into pathways based on the MataCyc database (Caspi et al., 2013). Further statistical analysis and data visualization were performed using STAMP version 2.1.3 (Parks et al., 2014).

### Comparison of taxonomic classification between FL-16S and V3V4-16S amplicon sequencing

The V3V4-16S amplicon sequences were generated by the previous report (Bunyoo et al., 2022). Briefly, the samples consisted of *L. punctata* cultivated under nutrient-deficient conditions for two weeks (*n* = 5). Sample collection, cultivation conditions, and DNA extraction were performed as previously described (Bunyoo et al., 2022). The library was prepared by amplifying the V3-V4 region of 16S rRNA gene using primers 341F (5-CCTAYGGGRBGCASCAG-3) and 806R (5-GGACTACNNGGGTATCTAAT-3) (Yu et al., 2005). Pair-end sequencing (2 × 250 bp) was performed using an Illumina Novaseq 6000 platform at NovogeneAIT Genomics Singapore Pte. The raw sequences were deposited in GenBank under BioProject number PRJNA888649.

The pair-end reads were processed using the package DADA2 in R (version 4.1.1) according to the DADA2 pipeline tutorial (1.16) (https://benjjneb.github.io/dada2/tutorial.html). The DADA2-formatted Silva version 138.1 database was used for taxonomic classification, with chloroplast and mitochondria reads removed from the final feature table. The taxonomic resolution and alpha diversity between FL-16S and V3V4-16S amplicon sequencing were compared.

### *In silico* PCR using FL-16S and V3V4-16S primers

To assess primer-associated bias toward preferential binding to certain taxa during PCR amplification, FL-16S and V3V4-16S (341F and 806R) primer sequences used in the previous study (Bunyoo et al., 2022) were aligned to the non-redundance SILVA Reference database (SSU Ref NR99 released 138.1). The alignment was carried out using TestPrime 1.0 (Klindworth et al., 2013) with zero mismatch allowed. For each primer pair, overall coverage was computed which indicated the eligible sequences that were long enough to cover both forward and reverse primers. From these eligible sequences, fractions of match/mismatches to the primers were calculated. The matched sequences indicated the sequences which can match perfectly with both forward and reverse primers. The fractions of match sequences were computed for all taxa.

### Statistical analysis

The differential abundance of microbial communities between natural and stress conditions was assessed using a negative binomial model (Wald test) implemented in DESeq2 version 1.38.3 (Love, Huber & Anders, 2014). The read counts were normalized using the estimateSizeFactors method (type = “poscounts”) prior to differential abundance analysis. The Pearson correlation coefficient (Pearson, 1895) was computed to compare the bacterial compositions between mock community samples and the theoretical composition. These statistical analyses were conducted with R (version 4.1.1; R Core Team, 2021). The differential abundance of predicted metabolic pathways of metagenomes between natural and stress conditions was analyzed using STAMP version 2.1.3 (Parks et al., 2014) by Welch’s *t*-test with a confidence interval of 0.95 (Welch, 1947). The *p*-value obtained from multiple pairwise testing was adjusted by Holm’s sequential Bonferroni method (Holm, 1979). The adjusted *p*-value <0.05 was considered statistically significant. Visualization of microbial composition was conducted by ggplot2 (Wickham, 2016) in R.

## Results

### Full-length 16S rRNA sequence data

A total of 324,266 demultiplexed CCS reads was obtained from all samples (*n* = 15) with a median of 12,125 reads (Table S1) as a result of FL-16S amplicon sequencing of natural-growing and stressed *L. punctata* using long-read sequencing. After removal of chloroplast and mitochondrial contamination, a total of 81,817 reads (25%) remained. The number of remaining reads per sample ranged between 99 to 11,055 reads with a median of 4,607 (Table S1). Of those, sample PreL_2 (99 reads) was dropped out due to insufficient read for analysis (Table S1). Subsequently, the processed reads obtained from 14 samples including *L. punctata* under natural condition (NC; *n* = 4), *L. punctata* under nutrient-deficient conditions (stress condition; SC; *n* = 5) and the ambient water (AW; *n* = 5) were used for investigation of microbiomes.

The highest contamination of chloroplast reads was observed in NC (85.9%), while SC had an average proportion (42.3%), and AW had less than 0.1% (Fig. S1). Contamination by mitochondrial reads was less than 0.1% across all samples (Fig. S1). After removal of plastid (chloroplast and mitochondria) reads, a total of 447 ASVs were detected across all samples. The rarefaction curves between observed ASVs and sampling depth plateaued in almost samples (Fig. S2). Sample metadata and features were displayed in Tables S2 and S3, respectively.

### Accuracy of PacBio sequencing

**Figure 1:**
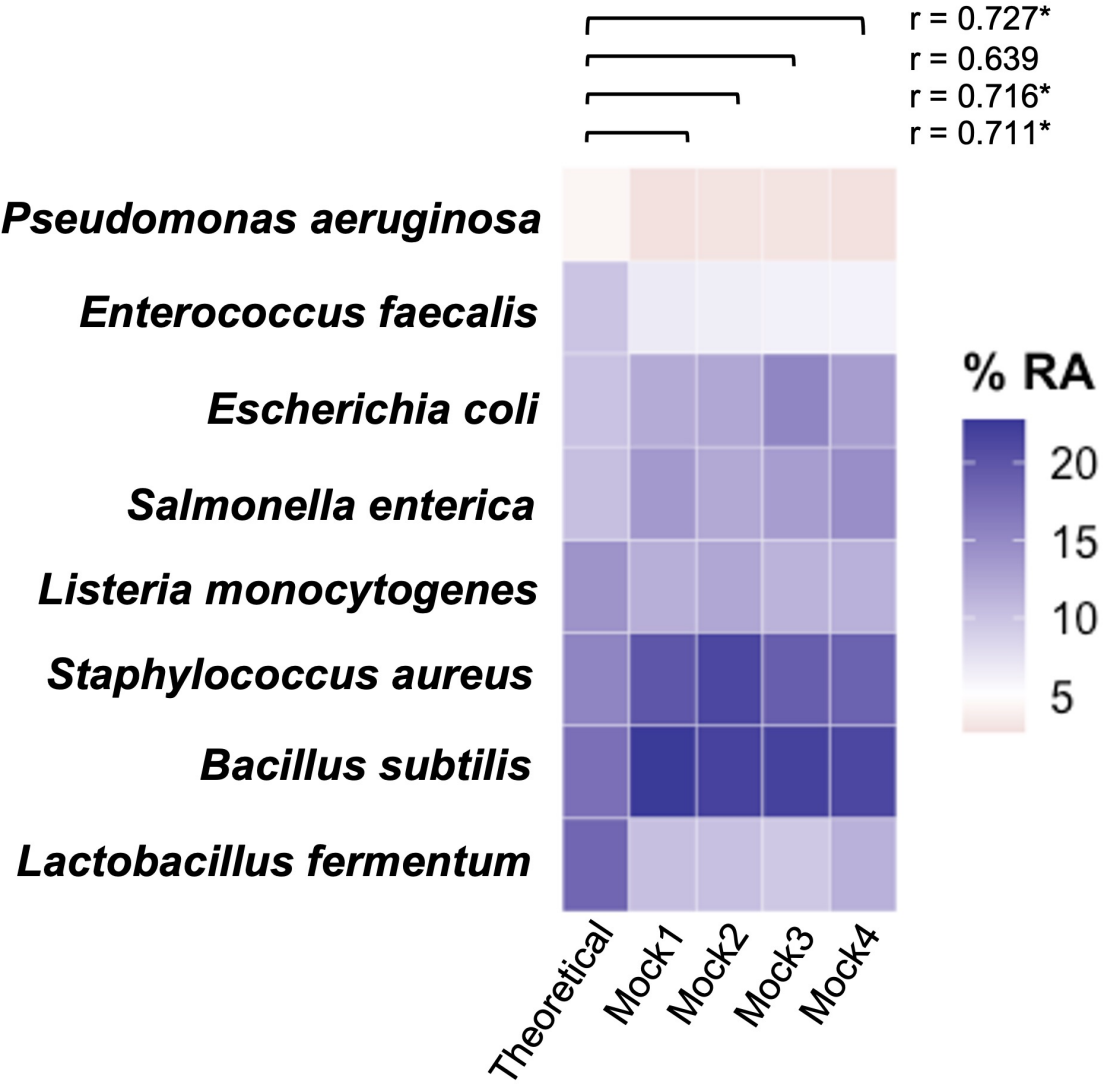
Relative abundance (RA; %) of mock bacterial community utilized as control. The Pearson correlation coefficient (*r*) between observed RA of each sample and theoretical RA was computed. Asterisks indicate significant correlation (*p*-value < 0.05).

To evaluate the accuracy of PacBio sequencing, mock bacterial communities were submitted along with each sequencing run. Overall, 62,836 CCS reads were obtained from mock samples (*n* = 4). Of these, 17 amplicon sequence variants (ASVs) were detected among mock samples (Table S4). Most reads (62,767 of 62,836 reads; 99.9%) could be aligned to mock reference sequences with percentage of identity of more than 99.5%, while 78% (49,100 of 62,836 reads) displayed 100% identity. The relative abundance (RA) of bacterial species detected in mock samples was further compared to the theoretical RA. Three out of four samples, including Mock1, Mock2, and Mock4 revealed significant correlation with the theoretical proportions: correlation coefficients (*r*) of 0.711, 0.716, and 0.727 (*p*-value <0.05), respectively (Fig. 1), while Mock3 displayed no significant correlation with the theoretical projection (*r* = 0.639; *p*-value = 0.087) (Fig. 1).

### Alteration of *L. punctata*-associated microbiome under nutrient-deficient condition

**Figure 2:**
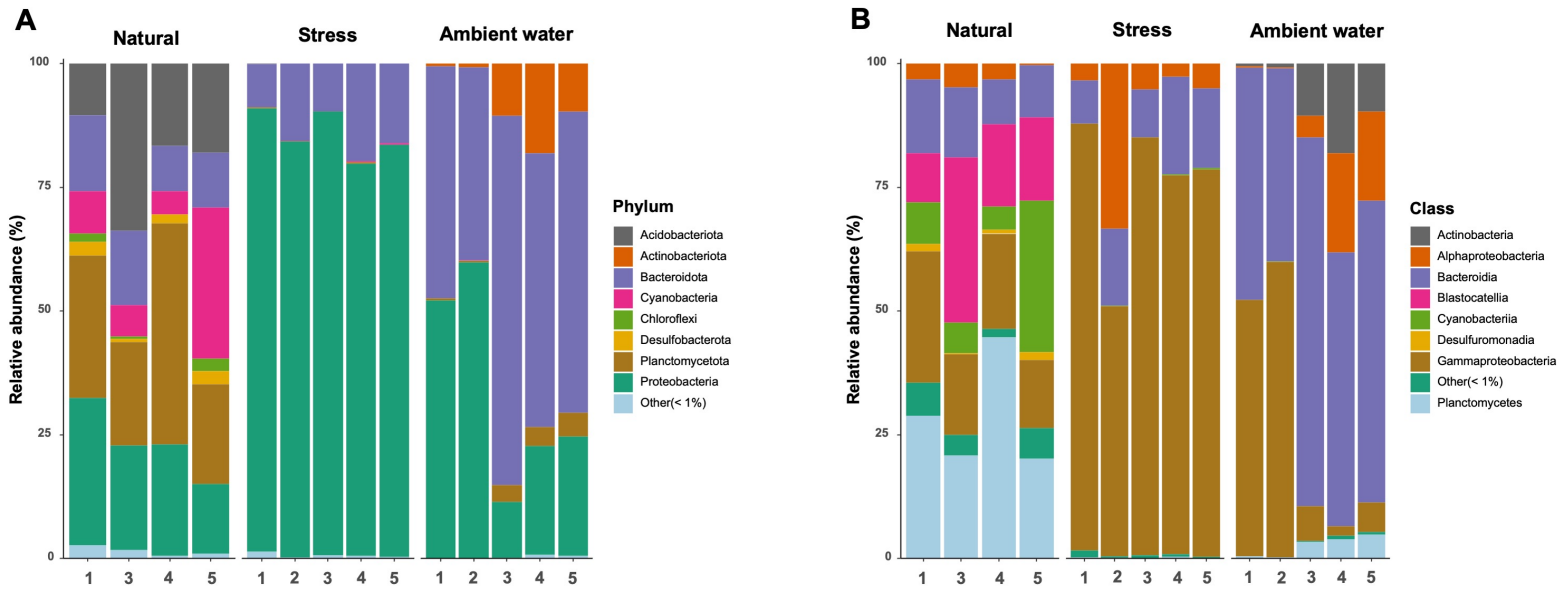
Microbial composition detected in each sample of microbiomes of *Landoltia punctata* under natural, stress (nutrient-deficient) conditions, and ambient water that classified at phylum (A) and class (B) levels.

Microbiomes of *L. punctata* in NC, SC, and AW were classified to taxonomic levels through their FL-16S sequences. The most abundant phyla in NC belonged to Planctomycetota and Proteobacteria with median RA of 25.3% and 21.8%, respectively (Fig. 2A; Table S5), followed by the phylum Acidobacteriota, Bacteroidota, Cyanobacteria, and Desulfobacterota with a series of median RA of 17.5%, 12.8%, 7.5%, and 2.3%, respectively (Fig. 2A). SC displayed the most prominent phylum of Proteobacteria followed by Bacteroidota with median RA of 84.1% and 15.7%, respectively (Fig. 2A; Table S5). Conversely, around half of the microbial community (55.4% median RA) in AW belonged to the phylum Bacteroidota followed by Proteobacteria, Actinobacteriota, and Planctomycetota, with median RA of 24.0%, 9.7%, and 3.4%, respectively (Fig. 2A; Table S5).

At class level, microbial communities of *L. punctata* in NC were mostly associated with Planctomycetes (phylum Planctomycetota; 25.3% median RA) followed by Gammaproteobacteria (phylum Proteobacteria; 17.9% median RA) and a lesser amount of Alphaproteobacteria (phylum Proteobacteria; 3.2% median RA) (Fig. 2B). On the other hand, Gammaproteobacteria appeared to dominate the microbiomes of *L. punctata* in SC (78.2% median RA) (Fig. 2B; Table S6). Microbiome of AW were associated primarily with the class Bacteroidia (phylum Bacteroidota; 55.2% median RA) that had a lower proportion in both NC and SC (Fig. 2B; Table S6).

**Figure 3:**
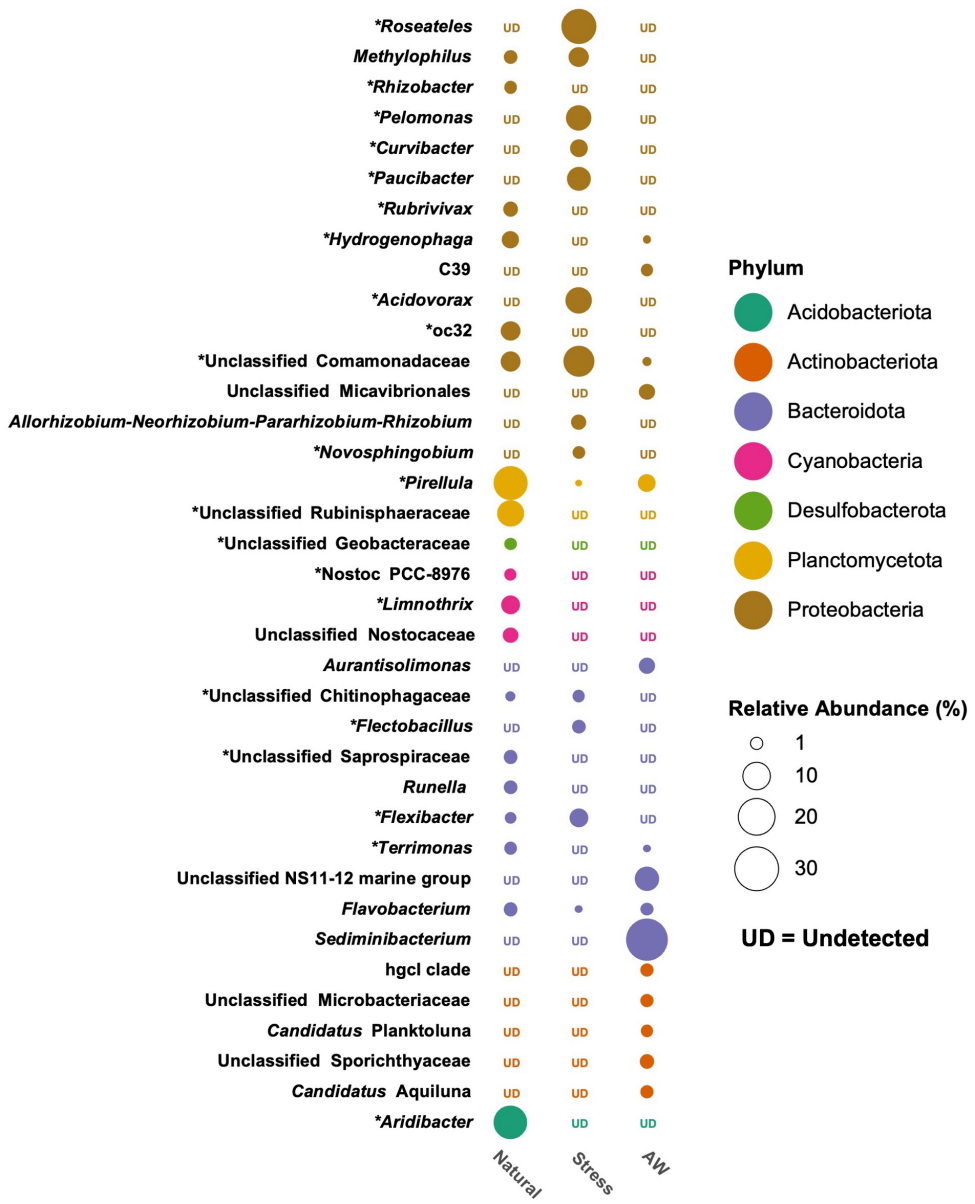
Comparison of microbial communities associated with *Landoltia punctata* under natural, stress (nutrient-deficient) conditions, and ambient water (AW). The microbial communities were classified at genus level and the abundance was displayed by median of relative abundances. An asterisk (*) indicates a significant difference between natural and stress conditions. UD, undetectable.

By using FL-16S sequences, around 77% of processed CCS reads were instantly classified at the genus level. Overall, 108 genera were detected across all samples. In NC, the genera *Pirellula* (phylum Planctomycetota) and *Aridibacter* (phylum Acidobacteriota) were detected in similar proportions (about 17.1% and 16.8%, respectively), followed by *Limnothrix* (phylum Cyanobacteria), *Hydrogenophaga* (phylum Proteobacteria), *Rubrivivax* (phylum Proteobacteria), and *Flavobacterium* (phylum Bacteroidota) (3.9%, 3.1%, 2.1%, and 1.6%, respectively (Fig. 3; Table S7). Members of the Proteobacteria mostly dominated in SC including the genus *Roseateles* (17.9%), followed by *Acidovorax*, *Pelomonas*, *Paucibacter*, and *Methylophilus* (9.2%, 8.2%, 7.2%, and 4.5%, respectively (Fig. 3; Table S7). Conversely, the genus *Sediminibacterium* (phylum Bacteroidota) was mostly found in AW (27.6%) followed by *Pirellula* (phylum Planctomycetota), *Aurantisolimonas* (phylum Bacteroidota) and *Candidatus* Aquiluna (phylum Actinobacteriota) (3.3%, 2.5%, and 1.4%, respectively (Fig. 3; Table S7).

Notably, the proportions of *Pirellula* and *Aridibacter*, predominantly found in NC, were reduced in SC. By contrast, the proportions of Proteobacteria such as *Roseateles*, *Acidovorax*, *Pelomonas*, and *Paucibacter* as well as members of the phylum Bacteroidota, including *Flectobacillus* and *Flexibacter*, were significantly increased (Fig. 3).

### Species detection of *L. punctata*-associated microbiomes

Of the total 447 ASVs detected across all samples, only 16% (72 of 447 ASVs) could be classified to species level using SILVA (version 138.1) as reference database. Therefore, the ASVs were further analyzed by aligning against GenBank 16S rRNA reference sequences (type strain) using BLASTN with percentage of identity above 97% and *E*-value cut-off at 1^e−10^. Consequently, approximately 57% of (255/447 ASVs) could be classified at species level (Table S8). The multiple ASVs assigned to the same species collapsed together.

**Figure 4:**
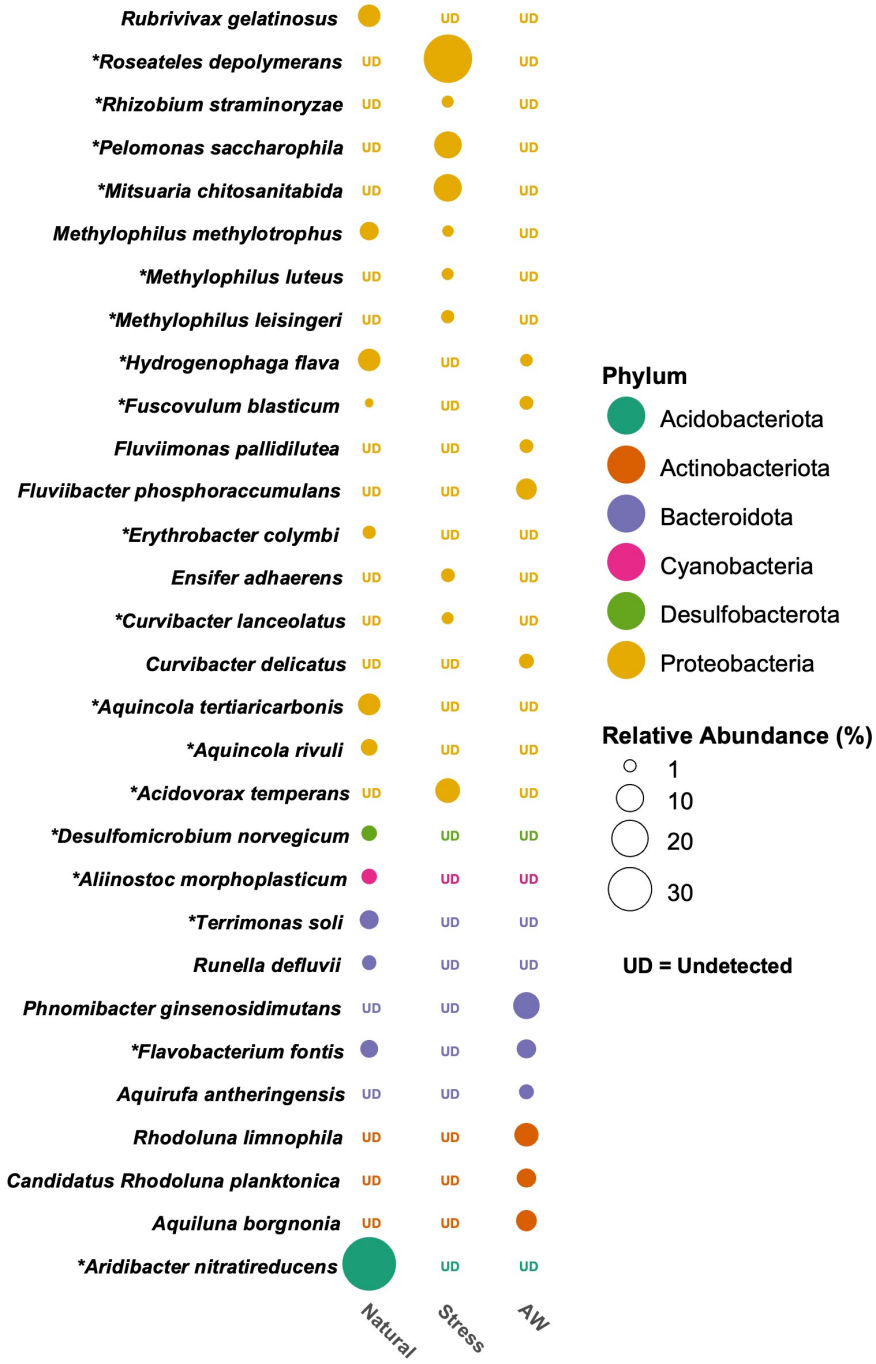
Comparison of microbial communities associated with *Landoltia punctata* under natural, stress (nutrient deficient) conditions, and ambient water (AW). The microbial communities were classified at species level and the abundance was displayed by median of relative abundances. An asterisk (*) indicates a significant difference between natural and stress conditions. UD, undetectable.

Thirty-one high-abundance species (each with >1% relative abundance) were identified among the samples (Table S9). Of NC microbiomes, *Aridibacter nitratireducens* was the most abundant species (50.9% median RA) followed by *Rubrivivax gelatinosus*, *Aquincola tertiaricarbonis*, *Hydrogenophaga flava*, *Terrimonas soli, Methylophilus methylotrophus*, and *Flavobacterium fontis* with median RAs of 6.4%, 6.3%, 6.1%, 4.1%, 3.9%, and 3.4% respectively (Fig. 4, Table S9). In SC, the proportions of *Roseateles depolymerans* (38.8%), *Pelomonas saccharophila* (10.4%)*, Acidovorax temperans* (8.2%), *Mitsuaria chitosanitabida* (11%), *Methylophilus leisingeri* (1.4%), *Curvibacter lanceolatus* (1.1%), and *Rhizobium straminoryzae* (1.1%) were significantly enhanced whereas *Ensifer adhaerens* (1.5%) was also detected but not significantly enhanced (Fig. 4, Table S9). By contrast, members of the Bacteroidota: *Phnomibacter ginsenosidimutans*, *Aquirufa antheringenis* and members of the Actinobacteriota: *Rhodoluna limnophila* and *Aquiluna borgnonia* that dominated in AW with respective median RAs of 9.9%, 2.0%, 7.3%, and 5.5% were found rarely in *L. punctata* microbiomes (Fig. 4, Table S9).

### Functional prediction of *L. punctata*-associated microbiome

**Figure 5:**
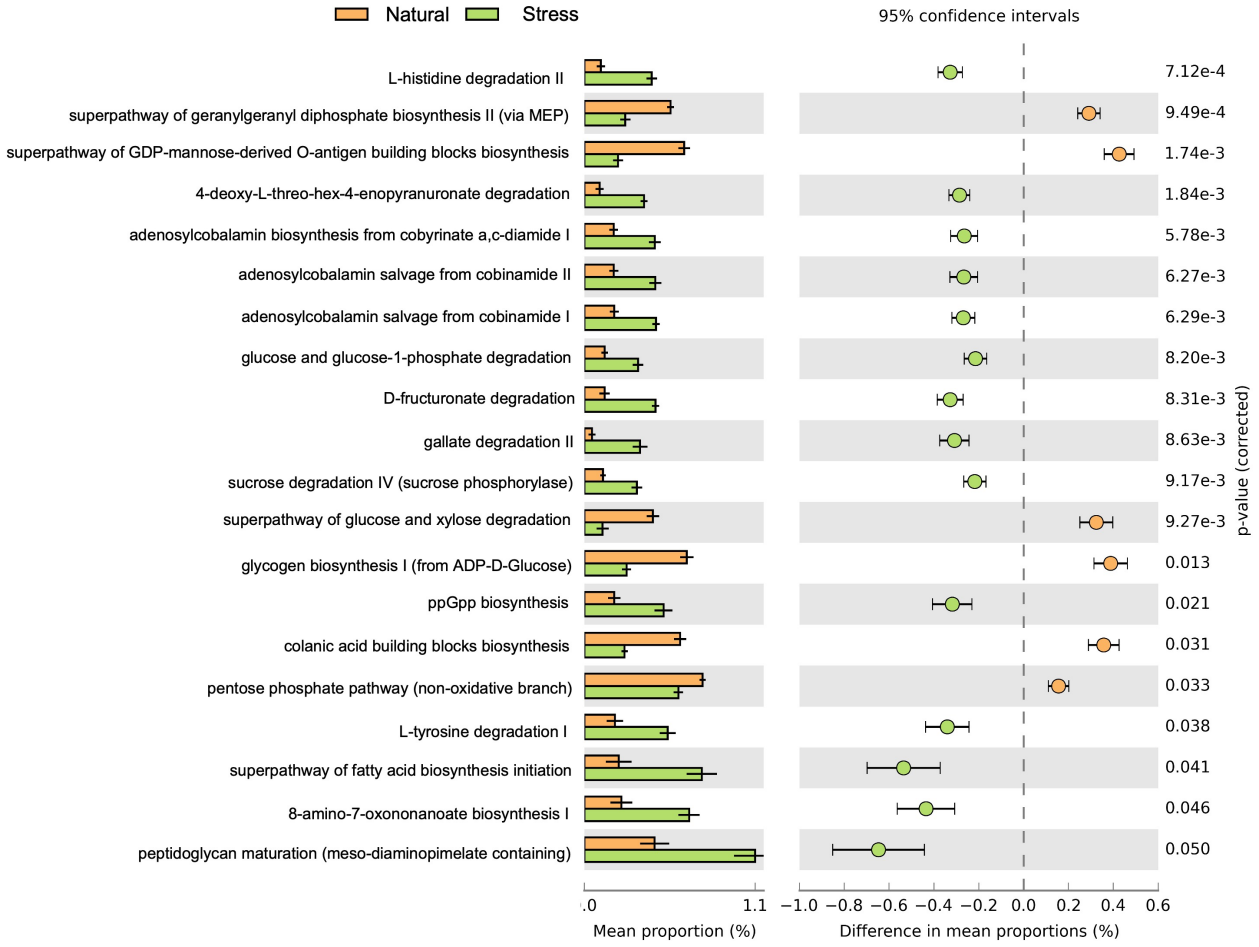
Predicted pathways of *Landoltia punctata*-associated microbiomes that were significantly different in natural and stress (nutrient-deficient) conditions. The bar plot indicates mean abundance of metabolic pathways in each condition along with the associated confidence interval of the effect size and *p*-value.

Based on the MetaCyc database, 351 metabolic pathways were predicted across samples of NC, SC, and AW (Table S10). The functional pathway abundances of microbiomes from different environmental conditions were clearly distinguishable using principal component analysis (PCA; Fig. S3). Among these, 20 pathways were significantly different between NC and SC (Fig. 5). In NC, enriched metabolic pathways included carbohydrate biosynthesis (*e.g.*, the superpathway of GDP-mannose-derived biosynthesis of O-antigen building blocks and biosynthesis of colanic acid building blocks), glycan biosynthesis (*e.g.*, glycogen biosynthesis I (from ADP-D-glucose)), pentose phosphate pathway, secondary metabolite biosynthesis (*e.g.*, superpathway of geranylgeranyl diphosphate biosynthesis II (*via* MEP)) (Fig. 5). Conversely, the main enriched pathways in SC related to degradation/utilization/assimilation: amino acid degradation (*e.g.*, L-tyrosine degradation I and L-histidine degradation II), carbohydrate degradation (*e.g.*, glucose and glucose-1-phosphate degradation and sucrose degradation IV (sucrose phosphorylase)), and secondary metabolite degradation (*e.g.*, D-fructuronate degradation). The pathways involved in biosynthesis were also significantly enriched in SC including cofactor and vitamin (*e.g.*, adenosylcobalamin salvage from cobinamide and adenosylcobalamin biosynthesis from cobyrinate a,c-diamide), cell structure (*e.g.*, peptidoglycan maturation (meso-diaminopimelate containing)), and metabolic regulator (*e.g.*, ppGpp) (Fig. 5).

### Performance of taxonomic resolution using FL-16S sequence data

**Figure 6:**
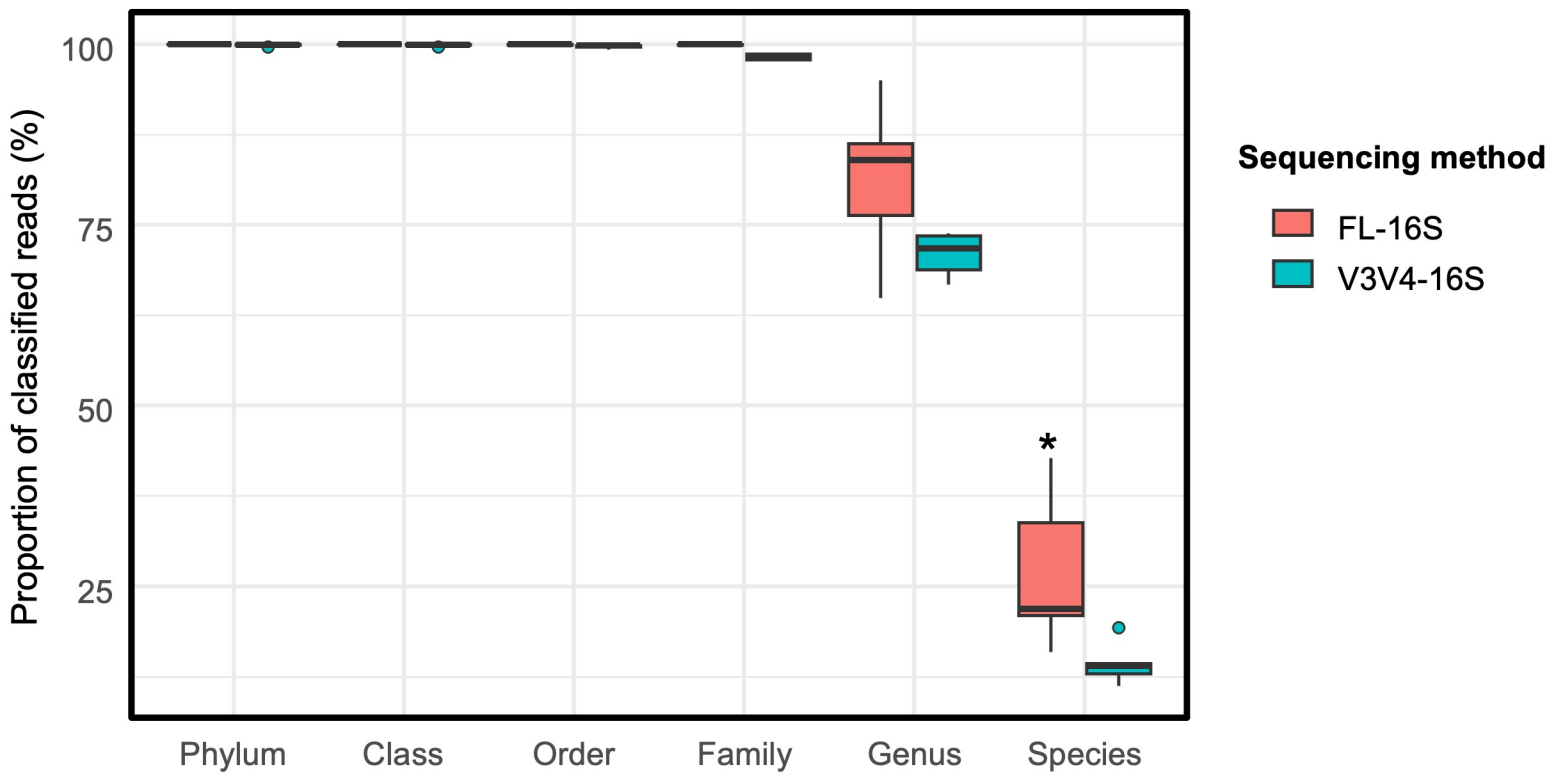
Proportions of sequence reads from full-length 16S rRNA gene (FL-16S) and V3V4 region of 16S rRNA gene (V3V4-16S) derived microbiomes of *Landoltia punctata* classifying at taxonomic levels. Asterisks (*) indicate significant different based on Wilcoxon rank-sum test, *p*-value <0.05.

To investigate the performance of taxonomic classification of *L. punctata* microbiomes at different levels, the FL-16S amplicon sequencing data (approximately 1,500 base pairs) obtained from PacBio sequencing were compared with the previous Illumina short-read V3V4-16S amplicon sequences (approximately 400 base pairs) (Bunyoo et al., 2022). To minimize bias introduced by magnitude of sample depths, samples of *L. punctata* in SC (*n* = 5) were trimmed to an even number of 5,000 reads prior to comparison. All sequences obtained from FL-16S and V3V4-16S were classified into phylum, class, order, and family levels at around 98.0–99.0% (Fig. 6). Of note, 81.3% and 27.1% of respective genus and species resolution were taxonomically classified from FL-16S, whereas 70.9% and 14.3% of respective genus and species levels were assigned from V3V4-16S (Fig. 6).

Overall, FL-16S amplicon sequencing classified 18 phyla across all samples, showing a similar taxonomic composition to that obtained with V3V4-16S, except for the absence of the phylum Planctomycetota. The phylum Planctomycetota which was abundant in NC detected by FL-16S (Fig. 2A), was rarely detected using V3V4-16S (Fig. S4). However, the total number of phyla classified using V3V4-16S was higher than that for FL-16S. Seven phyla were detected exclusively in the V3V4-16S dataset. However, these phyla were mostly rare, each accounting for less than 1% of the relative abundances. To compare richness and evenness of microbial communities obtained from two sequencing methods, alpha diversity, including Shannon and Simpson indices, were analyzed. The richness and evenness value from V3V4-16S dataset were significantly higher than those obtained using the FL-16S method (Fig. S5; Table S11).

### Evaluation of primers-associated biases

The FL-16S and V3V4-16S primer sequences were aligned to the non-redundant SILVA reference database to determine primer-associated biases that may occur during PCR amplification. These sequences were identified as eligible sequences that matched both forward and reverse primers perfectly. The fraction of matched sequences in major phyla detected across duckweed and ambient water samples included Acidobacteriota, Actinobacteriota, Bacteroidota, Desulfobacterota, Firmicutes, Planctomycetota, and Proteobacteria were selected for evaluation.

**Figure 7:**
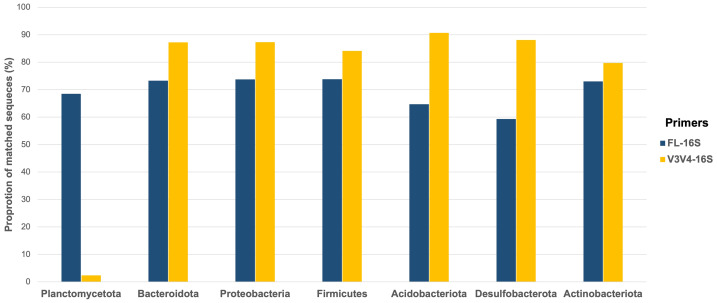
Evaluation of primer-associated bias toward preferential binding to certain phyla by *in silico* PCR. The FL-16S and V3V4-16S primer sequences were aligned to non-redundance SILVA reference database (SSU Ref NR99 released 138.1). The proportion of matched sequences indicated the fraction of eligible sequences (accession sequences that were long enough to analyze) which perfectly matched both forward and reverse primers.

Overall, the V3V4-16S primers had higher rates of matching to the eligible sequences of most phyla (except phylum Planctomycetota) than the FL-16S primers (Fig. 7). The V3V4-16S primers displayed the highest rate of matching to the phylum Acidobacteriota, (90.7%), followed by phyla Desulfobacterota (88.1%), Proteobacteria (87.3%), Bacteroidota (87.2%), Firmicutes (84.1%), and Actinobacteriota (79.7%); whereas phylum Planctomycetota had the lowest matching rate (2.4%; Fig. 7).

The FL-16S primers displayed the highest matching rate to the phylum Actinobacteriota, Bacteroidota, Firmicutes, and Proteobacteria with proportion of matched sequence around 73%, followed by phyla Planctomycetota (68.4%), Acidobacteriota (64.1%), and Desulfobacterota (59.3%) (Fig. 7).

## Discussion

In the previous study, the microbial communities associated with four species of duckweed grown under natural and nutrient-deficient conditions were investigated using V3V4 16S rRNA amplicon sequencing (Bunyoo et al., 2022). The findings revealed a potentially beneficial core of microbiomes that persisted in association with duckweed under stress. However, the microbial profiles in that study could not be classified at species level due to the use of partial 16S rRNA sequencing data. In this study, the most resilient species under stress conditions in the previous work, *L. punctata*, (data not shown) was thus selected for investigation of its associated microbiomes at the species level using full-length 16S rRNA amplicon sequencing.

Though PacBio CCS generates complete sequences of 16S rRNA genes, the raw data obtained from plant microbiome samples were highly contaminated with chloroplast and mitochondria sequences. After removal of these contaminations, only 25% reads (81,817) remained. The remaining reads per sample had a median of 4,670 and any sample contaminated by up to 99% of all sequences could be used for further analysis (Fig. S1; Table S1). Furthermore, the primers aligned to the chloroplast sequence of *L. punctata*. Both FL-16S and V3V4-16S primer pairs displayed perfect alignment with the chloroplast sequence (data not shown). Therefore, these primers should be improved further for duckweed 16S rRNA amplicon sequencing studies. Additionally, the proportion of chloroplast reads from samples under stress conditions was lower than those of natural conditions. This suggested that the exposure to stress affected the chlorophyll content of duckweed. Currently, there are several methods concerning primer design to prevent the amplification of chloroplast and mitochondria fractions in metagenome PCR reactions. These discriminate primers are specific to the bacterial DNAs but not to those of plant plastids (Chelius & Triplett, 2001; Bodenhausen, Horton & Bergelson, 2013). Another technique is a PCR clamp/blocking oligo method that uses probes specific to chloroplast sequences to prevent amplification (Giangacomo et al., 2021). All techniques could effectively reduce chloroplast contamination but required complicated steps that resulted in significant bias of the PCR reactions that led to inaccurate classification of the microbiome results (Giangacomo et al., 2021).

In this study, the total number of sequences obtained for each sample was less than expected due to plastid contamination. Therefore, the raw CCS reads were pre-filtered using a minimum number of CCS passes (three passes) prior to analysis. Though higher stringency of pre-filtering by increasing the number of CCS passes decreased the sequence error rates, it significantly reduced the number of raw reads (Wagner et al., 2016; Johnson et al., 2019). To evaluate the accuracy of sequence data, mock bacterial communities were utilized, which showed that 78% of the total reads were perfectly matched with the mock 16S reference sequences indicating no sequence errors. Furthermore, most erroneous reads could be aligned to mock reference sequences with percentages of identities of more than 99.5%. This percentage of identity is greater than the 97% threshold of identity for species discrimination (Schloss et al., 2016). The results suggest that the accuracy of sequencing is sufficient to discriminate bacteria at the species level. Furthermore, most mock samples revealed significant correlation to the theoretical predictions (Fig. 1).

Microbiomes of NC *L. punctata* of this study had Planctomycetota, Proteobacteria, and Bacteroidota as the most predominant phyla, similar to those of previous reports (Acosta et al., 2020; Inoue et al., 2022). In general, members of the Planctomycetota are key taxa contributing to global carbon and nitrogen cycles in aquatic and terrestrial environments (Wiegand, Jogler & Jogler, 2018). They are also in root-associated microbiomes that are beneficial for plant growth and stress tolerance (Lei et al., 2023; Tang et al., 2023; Tebele, Marks & Farrant, 2023). Proteobacteria and Bacteroidota were dominant phyla in plant phyllospheres (Trivedi et al., 2020). Under stress conditions, Proteobacteria dominated in *L. punctata*-associated microbiomes (Fig. 2A). Proteobacteria are often found predominately in plant environments that can help promote growth during nutrient-deficient conditions (Trivedi et al., 2020). Such characteristics may be due to the high abundance of genes related to substrate transporters found in pangenome analyses of Proteobacteria isolated from plants (Levy et al., 2017).

The dominant genus of phylum Planctomycetota such as *Pirellula* was found mainly associated with NC *L. punctata*, followed by members of the phylum Acidobacteriota: *Aridibacter*, and members of the phylum Proteobacteria: *Rhizobacter* and *Methylophilus* (Fig. 3). Notably, *Pirellula* was also presented in the ambient water samples. However, several major phyla were found in the surrounding water, such as *Sediminibacterium*, *Aurantimonas*, and *Candidatus* Planktoluna were not associated with *L. punctata* (Fig. 3). The results supported the premise that duckweed selectively recruited those microbes that are available in the water environment (Acosta et al., 2020; Inoue et al., 2022).

In our previous report, members of genera *Allorhizobium-Neorhizobium-Pararhizobium-Rhizobium*, *Pelomonas*, *Roseateles*, and *Novosphingobium* were persistently detected in the stressed *L. punctata* samples, when V3V4-16S amplicon sequencing was employed (Bunyoo et al., 2022). Remarkably, these microbiomes could be classified using FL-16S at species resolution—namely *Rhizobium straminoryzae*, *Ensifer adhaerens, Pelomonas saccharophila*, and *Roseateles depolymerans* (Fig. 4) which are members of the Proteobacteria that reported previously as bacteria that are beneficial to plants and/or bioremediators (Suyama et al., 1999; Baliyan et al., 2022; Makino et al., 2022; Boonmak et al., 2023). In our previous study, the genera *Acidovorax* and *Novosphingobium*, specifically *Acidovorax kalamii* and *Novosphingobium subterraneum*, were identified as members of core microbiomes associated with *Spirodela polyrhiza* which helped promote the growth of duckweed in wastewater (Boonmak et al., 2023). Members of methanol-utilizing bacteria, *Methylophilus*, were also frequently detected in this work. They are mainly found in rivers, lakes, ponds, and plants, and have been established as pollutant-tolerantt bacteria suitable for bioremediation (De Marco et al., 2004; Doronina, Kaparullina & Trotsenko, 2014). Furthermore, *Methylophilus luteus* and *Rhizobium straminoryzae* previously reported as plant-associated microbes in coltsfoot and rice (Gogleva et al., 2010; Lin et al., 2014), were significantly enhanced in stressed *L. punctata* (Fig. 4).

*Pelomonas saccharophila*, a member of the family Comamonadaceae, was mostly found in water or muddy soil and had the ability to fix nitrogen using the *nif*H gene product (Xie & Yokota, 2005). It is also able to produce a plant hormone, indole-3-acetic acid (IAA) which has been proven as a plant growth promoting bacteria (PGPB) by enhancing biomass and the chlorophyll content of *Lemna minor* (Makino et al., 2022). A phototrophic bacterium, *Roseateles depolymerans*, a member of the family Sphaerotilaceae and, reported in this study was detected previously in fresh water and displayed a capability for bioremediation by producing hydrolytic enzymes for poly(hexamethylene carbonate) and bioplastic degradation (Suyama et al., 1999). Additionally, a genome analysis of *Acidovorax temperans* isolated from sewage water identified genes related to denitrification and phosphorus accumulation which could potentially enhance the efficiency of wastewater bioremediation (Araú & Anjos de, 2020). *Ensifer adhaerens*, a nitrogen-fixing bacterium acting as an obligate predator under nutrient limitation conditions (Rogel et al., 2001), was detected in this work. It was reported previously that members of this genus are PGPB. For example, *Ensifer* sp. SP4 promoted *S. polyrhiza* growth through enhancing nitrogen metabolism and photosynthesis (Toyama et al., 2022), *E. adhaerens* MSN12 improved growth rate and yield of chicken pea (Baliyan et al., 2022), and *E. adhaerens* OS3 demonstrated PGP traits under metal stress (Oves, Khan & Qari, 2017). Interestingly, certain strains of *E. adhaerens* are recognized as cobalamin (vitamin B_12_) producers and have been shown to be safe to use as food and feed additives (Zhao et al., 2019).

Functional predictions of *L. punctata*-associated microbiomes in NC, SC, and microbial communities in AW showed clear differences (Fig. S2). The results suggest that environmental conditions are a major factor determining the structure and function of microbial communities. In NC, enriched microbial functions were related to carbohydrate biosynthesis such as the superpathway for biosynthesis of GDP-mannose-derived O-antigen building blocks and for colanic acid building blocks (Fig. 5). O-antigen comprises units of lipopolysaccharides (LPS) which are major components of the outer membrane of Gram-negative bacteria (Caroff & Karibian, 2003). LPS is essential for the early recognition steps in rhizobium-legume interactions and root adhesion (Lerouge & Vanderleyden, 2002; Serrato, 2014). Genes involved in LPS biosynthesis in DAB, for example *Aquitalea magnusonii*, are responsible for the initial association with and fitness of duckweed (Ishizawa et al., 2022). Colanic acid, an exopolysaccharide produced by certain bacteria, plays an important role in formation of biofilms, benefiting cell adhesion and protection (Limoli, Jones & Wozniak, 2015). Secondary metabolite biosynthetic pathways such as the superpathway of geranylgeranyl diphosphate biosynthesis II (*via* methylerythritol phosphate (MEP)), was significantly enriched in NC *L. punctata* (Fig. 5). The MEP pathway is involved in biosynthesis of a variety of isoprenoid compounds in bacteria and plants that play important roles in protection against oxidative stress (Perez-Gil et al., 2024). These findings suggest that these microbial communities are likely to be an intact DAB which provide a beneficial function to natural-growing *L. punctata.*

Conversely, metabolic pathways related to carbohydrate and amino acid degradation were significantly enriched under nutrient-deficient conditions (Fig. 5), indicating that these microbes utilized carbohydrates and amino acids as their energy source for survival. Under carbon or nitrogen depletion, *Pseudomonas aeruginosa* consumes various amino acids as sole carbon or nitrogen sources (Kay & Gronlund, 1969). Moreover, metabolic regulator biosynthesis pathways for metabolic regulators such as ppGpp were enhanced (Fig. 5). Under carbon or nitrogen source starvation, intracellular ppGpp is triggered and accumulated (Wang, Grant & Laub, 2020) which, thereby, reshapes microbial metabolism by modulation of growth through transcription, translation, and the cell cycle (Ronneau & Hallez, 2019). Furthermore, ppGpp is involved in biofilm formation, virulence and antibiotics tolerance, that are crucial for microbial survival under various stressors (Ronneau & Hallez, 2019). Cofactor and vitamin biosynthesis pathways, such as salvage of adenosylcobalamin from cobinamide and adenosylcobalamin biosynthesis from cobyrinate a,c-diamide I were also enriched (Fig. 5). Notably, these pathways are related to cobalamin (vitamin B_12_) biosynthesis, which is exclusive to bacteria and archaea (Fang, Kang & Zhang, 2017). Recent studies have revealed that the presence of bioactive vitamin B_12_ in a variety of duckweed species is contributed by the associated bacteria (Acosta et al., 2024). Collectively, the predicted metabolic pathways of *L. punctata*-associated microbiomes suggest that these persistent microbial communities are adapted to survive under abiotic stress and may enhance the nutritional quantity of duckweed.

In this study, the proportion of FL-16S sequences classified at the genus and species levels was higher than those of V3V4-16S. The results indicate that full-length 16S rRNA genes provide better taxonomic resolution than partial sequences (Pootakham et al., 2017; Johnson et al., 2019; Jeong et al., 2021; Matsuo et al., 2021; Hale et al., 2024). By using V3V4-16S amplicon sequencing, members of the phylum Planctomycetota were detected rarely in the natural growing duckweeds (Bunyoo et al., 2022). On the contrary, in this work, a high abundance of the phylum Planctomycetota was detected in the FL-16S amplicon data. An assessment of primer-associated bias through *in silico* PCR revealed that the universal V3V4-16S primers exhibited the lowest matching rate for Planctomycetota members (Fig. 7). As a result, these primers likely failed to amplify members of Planctomycetota, which is dominant in aquatic environments (Wiegand, Jogler & Jogler, 2018). This finding underscores the importance of selective primers for metagenomic studies.

## Conclusions

This study demonstrates species-level profiling and putative functional insight into *L. punctata*-associated microbiomes under natural and stress conditions. The analysis of *L. punctata* samples here extended our previous study, where the microbiomes could only be classified at the genus level due to the use of partial V3V4 16S rRNA sequencing. Conversely, the full-length 16S rRNA amplicon sequencing data in this study identified the microbiomes associated with *L. punctata* at the species level. Furthermore, these findings highlight both the effectiveness and limitations of using partial *versus* full-length 16S rRNA amplicon sequencing. Isolation of key species associated with *L. punctata* and validation of their roles and potential benefits to duckweed through co-cultivation experiments are under investigation. Ultimately, the study will contribute to selection of beneficial species based on metagenomic data, facilitating the establishment of stable synthetic microbial communities to enhance biomass production, improve wastewater remediation efficiency, and/or increase their nutritional value for future food/feed sources.

##  Supplemental Information

10.7717/peerj.20648/supp-1 Supplemental Information 1 The proportion of plastid contamination sequences in each sample composed of *L. punctata* under natural condition (natural), nutrient deficient condition, (stress), and ambient water

10.7717/peerj.20648/supp-2 Supplemental Information 2 The rarefaction curves between observed ASVs and sampling depth

10.7717/peerj.20648/supp-3 Supplemental Information 3 Principal component analysis (PCA) plot of predicted metabolic pathways of * L. punctata* microbiomes under natural conditions (natural), nutrient deficient conditions (stress), and ambient water

10.7717/peerj.20648/supp-4 Supplemental Information 4 Microbial phyla detected in each sample of microbiomes of *Landoltia punctata* under natural, stress (nutrient-deficient), and ambient water conditions that were obtained by V3V4 16S rRNA sequencing

10.7717/peerj.20648/supp-5 Supplemental Information 5 Comparison of alpha diversity indices (Shannon and Simpson) between full-length (FL) and V3V4 16S rRNA (V3V4) sequencing Asterisks (*) indicate significant difference based on Wilcoxon rank-sum test, *p*-value ¡0.05.

10.7717/peerj.20648/supp-6 Supplemental Information 6 Number of sequences obtained from all samples

10.7717/peerj.20648/supp-7 Supplemental Information 7 Metadata in this study

10.7717/peerj.20648/supp-8 Supplemental Information 8 ASVs abundance and sequence reads of microbiomes of *Landoltia punctata* samples under natural, nutrient-deficient and ambient water

10.7717/peerj.20648/supp-9 Supplemental Information 9 ASVs abundance and sequence reads of standard microbial communities (Mock)

10.7717/peerj.20648/supp-10 Supplemental Information 10 Top abundance (>1% relative abundance) phyla with median of relative abundance (RA) of microbiomes of *Landoltia punctata* under natural, nutrient-deficient, and ambient water

10.7717/peerj.20648/supp-11 Supplemental Information 11 Top abundance (>1% relative abundance) class with median of relative abundance (RA) of microbiomes of *Landoltia punctata* under natural, nutrient-deficient, and ambient water

10.7717/peerj.20648/supp-12 Supplemental Information 12 Top abundance genera with relative abundance of microbiomes of *Landoltia punctata* under natural, nutrient-deficient, and ambient water

10.7717/peerj.20648/supp-13 Supplemental Information 13 The ASVs classified by aligning to GenBank 16S rRNA reference sequences (type strain) using BLASTN

10.7717/peerj.20648/supp-14 Supplemental Information 14 Top abundance species with relative abundance of microbiomes of *Landoltia punctata* under natural, nutrient-deficient, and ambient water

10.7717/peerj.20648/supp-15 Supplemental Information 15 Metabolic pathways and functions of microbiome in each sample predicted using PICRUSt2

10.7717/peerj.20648/supp-16 Supplemental Information 16 Alpha diversity indices were calculated from samples obtained using the FL-16S and V3V4-16S sequencing methods

## Additional Information and Declarations

### Data Availability

The following information was supplied regarding data availability: The raw sequence data generated in this study are available at National Center for Biotechnology Information (NCBI): PRJNA1016047.
