# Supplementary figures and images for: Species-level profiling of Landoltia punctata (duckweed) microbiome under nutrient stress using full-length 16S rRNA sequencing
Source: PeerJ. 2026 Feb 6;14:e20648. doi: 10.7717/peerj.20648 (PMC12884961; doi:10.7717/peerj.20648)

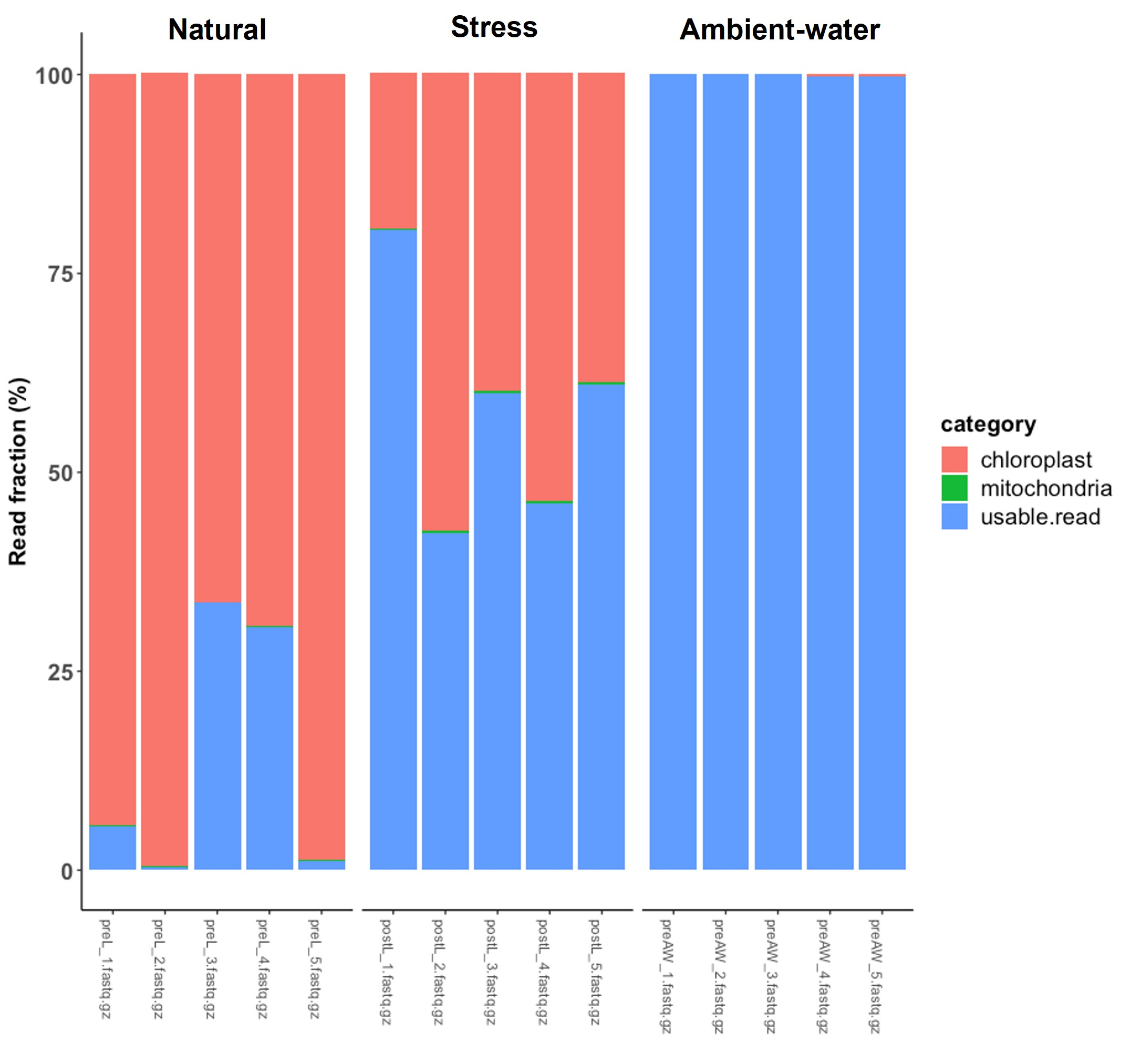

Supplement: Supplemental Information 1 [file peerj-14-20648-s001.png]

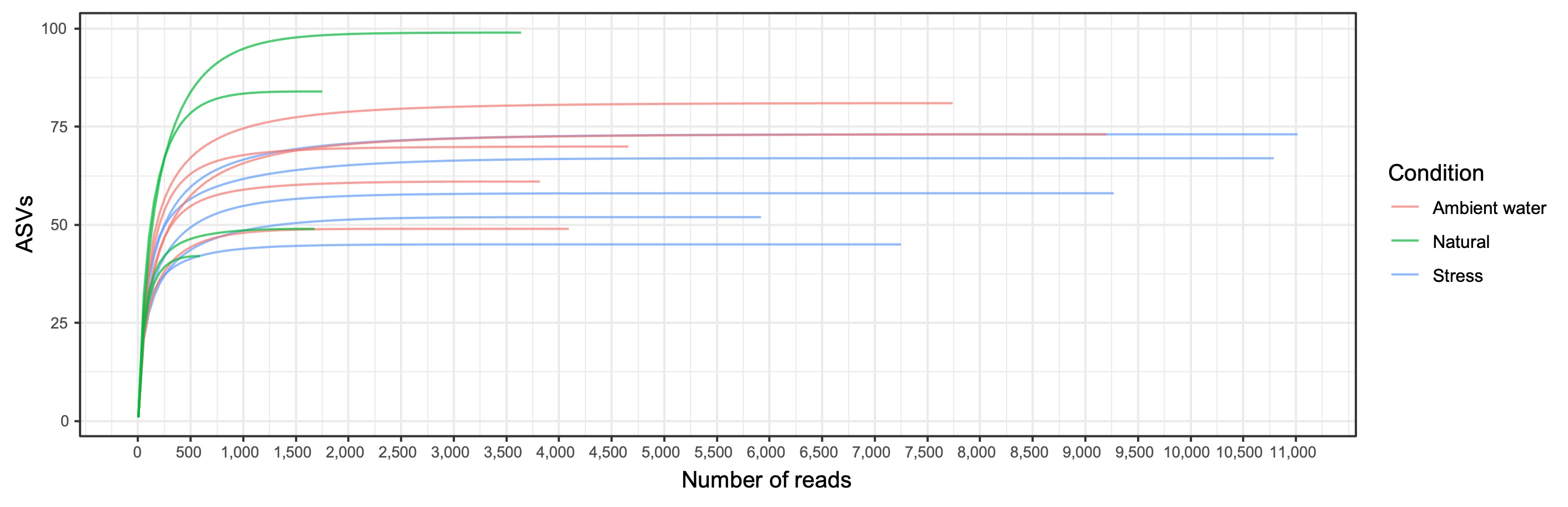

Supplement: Supplemental Information 2 [file peerj-14-20648-s002.png]

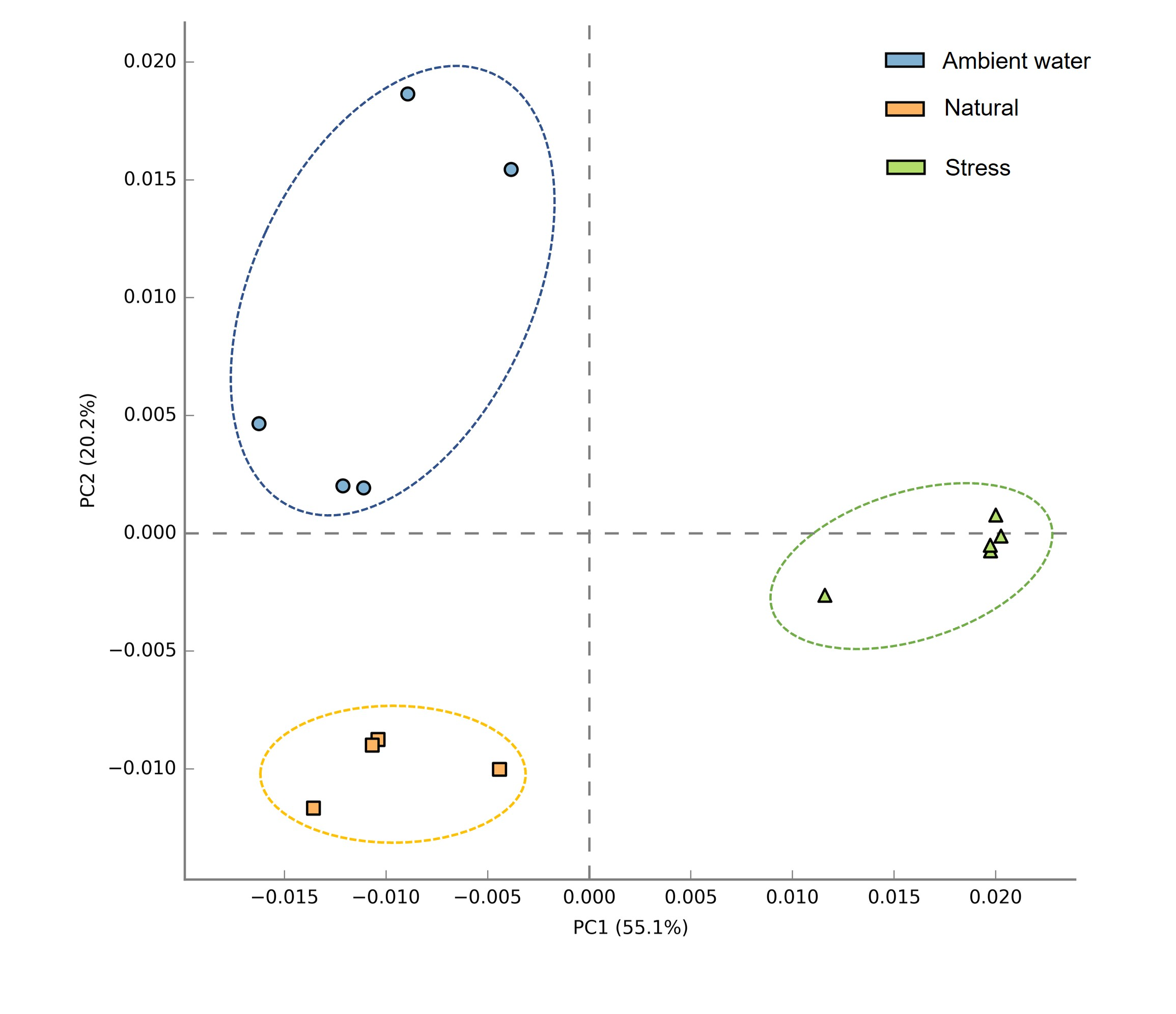

Supplement: Supplemental Information 3 [file peerj-14-20648-s003.png]

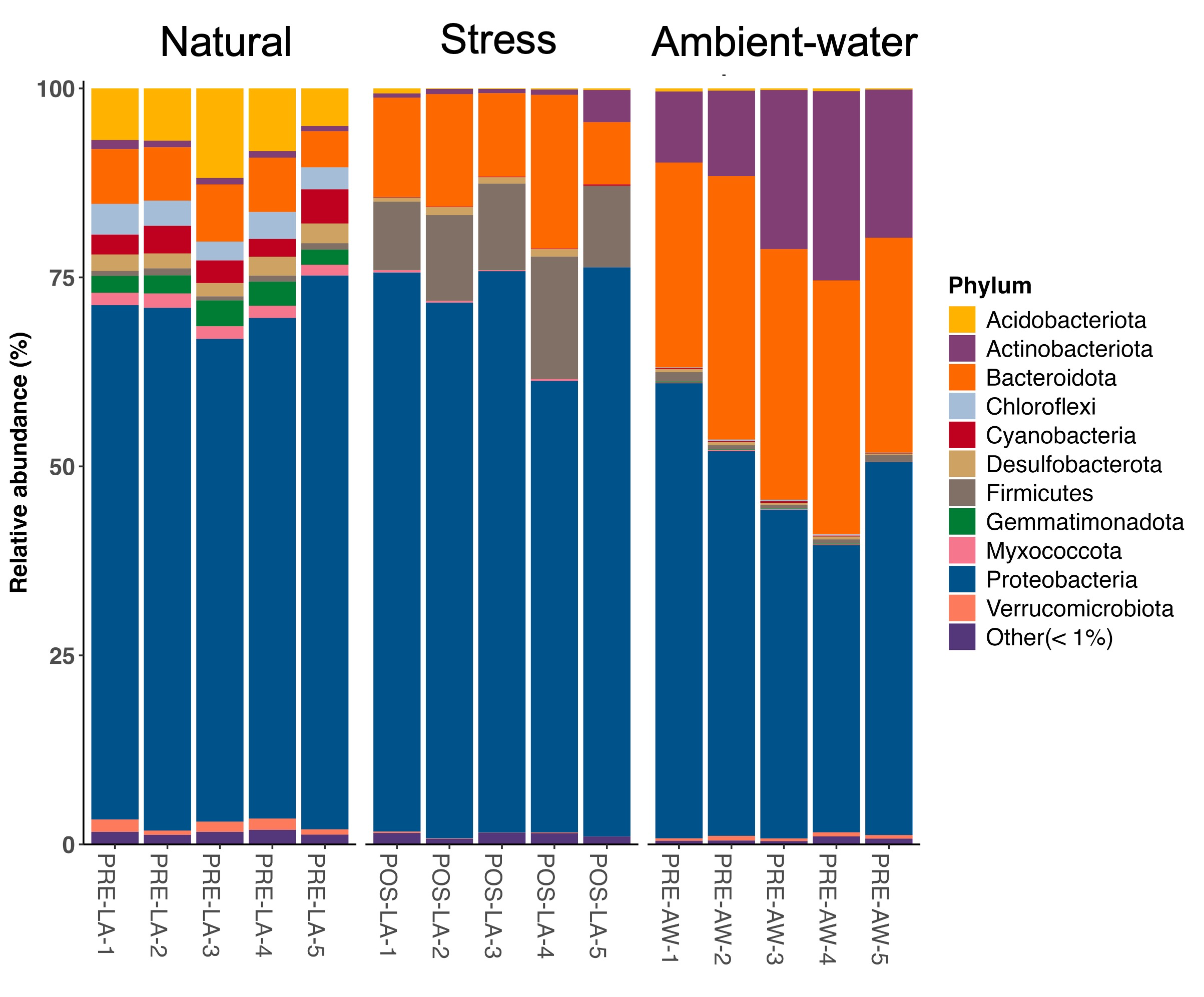

Supplement: Supplemental Information 4 [file peerj-14-20648-s004.png]

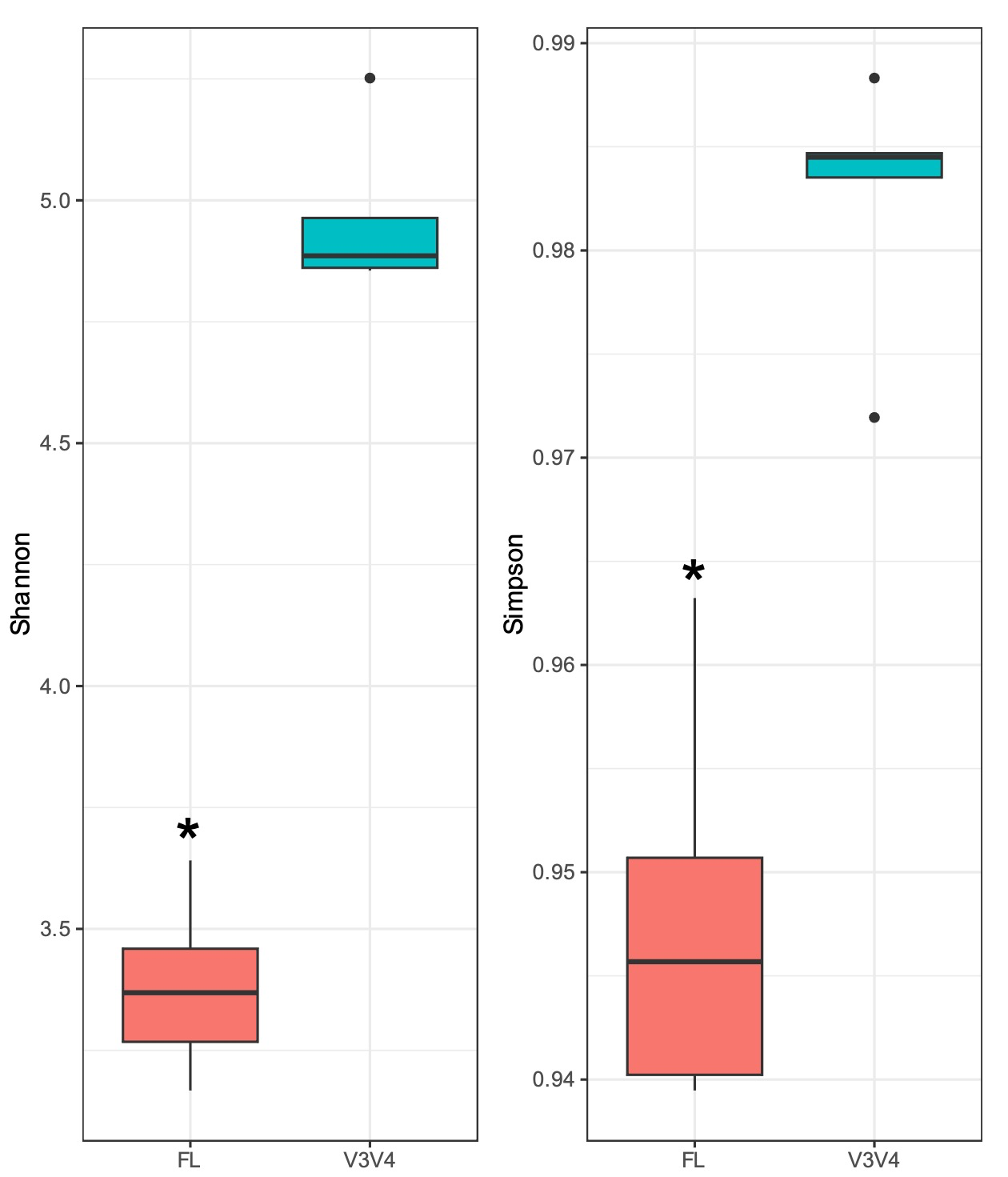

Supplement: Supplemental Information 5 — Asterisks (*) indicate significant difference based on Wilcoxon rank-sum test, p-value ¡0.05. [file peerj-14-20648-s005.png]
